# Melatonin Mitigates Lead-Induced Oxidative Stress and Modifies Phospholipid Profile in Tobacco BY-2 Suspension Cells

**DOI:** 10.3390/ijms25105064

**Published:** 2024-05-07

**Authors:** Agnieszka Kobylińska, Przemysław Bernat, Małgorzata Maria Posmyk

**Affiliations:** 1 Department of Plant Ecophysiology, Faculty of Biology and Environmental Protection, University of Lodz, Banacha 12/16, 90-237 Lodz, Poland; malgorzata.posmyk@biol.uni.lodz.pl; 2 Department of Industrial Microbiology and Biotechnology, Faculty of Biology and Environmental Protection, University of Lodz, Banacha 12/16, 90-237 Lodz, Poland; przemyslaw.bernat@biol.uni.lodz.pl

**Keywords:** BY-2 tobacco cells, lead, lipid peroxidation, melatonin, phospholipids, phospholipase, ROS

## Abstract

Many studies have shown that melatonin (an indoleamine) is an important molecule in plant physiology. It is known that this indoleamine is crucial during plant stress responses, especially by counteracting secondary oxidative stress (efficient direct and indirect antioxidant) and switching on different defense plant strategies. In this report, we present exogenous melatonin’s potential to protect lipid profile modification and membrane integrity in *Nicotiana tabacum* L. line Bright Yellow 2 (BY-2) cell culture exposed to lead. There are some reports of the positive effect of melatonin on animal cell membranes; ours is the first to report changes in the lipid profile in plant cells. The experiments were performed in the following variants: LS: cells cultured on unmodified LS medium—control; (ii) MEL: BY-2 cells cultured on LS medium with melatonin added from the beginning of culture; (iii) Pb: BY-2 cells cultured on LS medium with Pb^2+^ added on the 4th day of culture; (iv) MEL+Pb: BY-2 cells cultured on LS medium with melatonin added from the start of culture and stressed with Pb^2+^ added on the 4th day of culture. Lipidomic analysis of BY-2 cells revealed the presence of 40 different phospholipids. Exposing cells to lead led to the overproduction of ROS, altered fatty acid composition and increased PLD activity and subsequently elevated the level of phosphatidic acid at the cost of dropping the phosphatidylcholine. In the presence of lead, double-bond index elevation, mainly by higher quantities of linoleic (C18:2) and linolenic (C18:3) acids in the *log* phase of growth, was observed. In contrast, cells exposed to heavy metal but primed with melatonin showed more similarities with the control. Surprisingly, the overproduction of ROS caused of lipid peroxidation only in the stationary phase of growth, although considerable changes in lipid profiles were observed in the *log* phase of growth—just 4 h after lead administration. Our results indicate that the pretreatment of BY-2 with exogenous melatonin protected tobacco cells against membrane dysfunctions caused by oxidative stress (lipid oxidation), but also findings on a molecular level suggest the possible role of this indoleamine in the safeguarding of the membrane lipid composition that limited lead-provoked cell death. The presented research indicates a new mechanism of the defense strategy of plant cells generated by melatonin.

## 1. Introduction

Violent industrialization, urbanization and unlimited burning of fossil fuels carried out during the past few decades have given rise to serious problems of environmental contamination. Of the total ninety naturally present elements, fifty-three are considered heavy metals but only seventeen can be available to living cells and be important to plants. Increases in the levels of heavy metals in soils could be attributed to factors such as soil properties or different agricultural practices like the application of sludge to agricultural land [1]. Among the heavy metals, aluminium (Al), arsenic (As), cadmium (Cd), lead (Pb), mercury (Hg) and silver (Ag) have no known functions as nutrients [2]. According to the United States Environmental Protection Agency (EPA), heavy metals are on the list of priority pollutants. In terms of environmental risks, Pb, Hg and As are ranked as first, second and third, respectively, on the list of the US Agency for Toxic Substances and Disease Registry (ATSDR) [3,4,5,6].

Lead is present in the atmosphere as dust, fumes, mists, vapours and in the soil as minerals (PbCO_3_, PbS, PbSO_4_). The contamination of soils is one of the most serious problems due to the fact that this metal is cumulated there (sediment from atmospheric resources) and exhibits very high stability and a lack of biodegradability [7]. The accumulation of Pb in agricultural soil can lead to taking up the crops, then through the food chain expose animals and humans to possible adverse effects like multiple organ damage including kidneys, lungs, bones, etc. [8,9]. The uptake and bioaccumulation Pb in plants takes place either directly via the roots along with water or in part through absorption via shoots and foliage from the air [10]. The uptake of lead compounds is regulated by pH, particle size, and the cation exchange capacity of the soils, as well as by root exudation and other physico-chemical parameters. Lead toxicity causes a range of damages to plants from germination phase to yield formation; however, this is both plant developmental stage- and stressor concentration-dependent [11]. High Pb concentrations inhibit the activity of key enzymes, e.g., acid phosphatase, esterases, peroxidases, malic dehydrogenase, etc., by reacting with their sulfhydryl groups. Moreover, Pb disturbs plant water management and limits its mineral nutrition [12]. Excess Pb causes stunted growth, chlorosis and the blackening of the root system. Pb inhibits photosynthesis, upsets ionic balance, and changes hormonal status. Moreover, heavy metals including Pb encourage reactive oxygen species (ROS) generation, such as the superoxide anion-radical, singlet oxygen, hydrogen peroxide, and hydroxyl radical leading to oxidative stress in tissues. Mechanisms of negative influence of Pb also involve protein oxidation, a decrease in saturated fatty acid and an increase in unsaturated fatty acid contents in biomembranes and lipid peroxidation [13,14]. Lipid hydroperoxides, created as products of lipid peroxidation, can easily decompose into several reactive species, i.e., lipid alkoxyl radicals, aldehydes, e.g., malondialdehyde or lipid epoxides [15]. These products affect membrane structure and permeability. Moreover, they are toxic and active mutagens causing DNA damage and gene mutation [16]. Finally, Pb exposition can provoke signal transduction cascade towards cell death [17,18].

The sedentary lifestyles of plants have evolutionarily resulted in the exceptional metabolic flexibility of these organisms (among others secondary metabolism), which leads them to survive in changing environments. In any plant exposed to harmful factors, a quick and remarkable variation arises inside cells to increase their resistance or stress tolerance [19,20]. During plant acclimation to the harsh environment, different strategies (alternative metabolic pathways) are activated; however, biostimulants that change the metabolism before and *a priori* prepare the plant for stress are particularly useful. Melatonin (N-acetyl-5-methoxy-tryptamine) seems to have such properties. This indoleamine has been detected in numerous plant species [21]. It is considered a plant growth regulator because of its influence on the growth of whole plants, explants, the germination of seeds and the disruption in leaf senescence [22]. In addition, melatonin has been found to fortify plant tolerance to a range of abiotic stresses such as cold [23], copper [24], cadmium [25], lead [17,18], high temperature [26], salt [27], osmotic [28], drought stresses [29] and pathogen infection [30]. Our previous study revealed that melatonin also redirects carbohydrate metabolism during sugar starvation and induces gluconeogenesis to obtain basic energy substrates, which is another defense strategy [31]. The mechanisms of melatonin-mediated stress tolerance also involve the stimulating of antioxidant enzymes, the synthesis of glutathione and activating other antioxidants [23,32]. Moreover, the literature data indicate that the metabolites of melatonin, e.g., cyclic-3-hydroxymelatonin, 2-hydroxylmelatonin and N1-acetyl-N2-formyl5-methoxykynu-ramine (AFMK), also exhibit antioxidant activity [33,34,35]. These facts, together with melatonin’s small size and amphiphilic nature, make it particularly capable of translocating easily between cell compartments and protecting cell structures against oxidation.

It is well known that membrane lipids and lipid-derived molecules play a crucial role in the plant response to biotic and abiotic stresses [36]. Phospholipids (PLs), including primarily phosphatidylcholine (PC), phosphatidylethanolamine (PE), phosphatidylserine (PS), phosphatidylinositol (PI), phosphatidic acid (PA) and phosphatidylglycerol (PG), are considered to be the most sensitive in lipid fraction to alterations in the environment, resulting in ROS overproduction [37,38]. They constitute about 70% of all membrane lipids, and they are a part of plant cellular signalling similar to lipid-modifying enzymes, which, through lipid metabolism products, have important functions in adaptation to adverse growth conditions. Among them, phospholipase D (PLD) activity has been associated with a variety of environmental stresses responses, i.e., drought, freezing, wounding, phosphorus starvation or heavy metal (Cd, Ni, Cu and Hg) toxicity [39,40]. PLD hydrolyzes PC or PE to form PA; thus, the functions of PLD are often implemented through its enzymatic product, PA, which is currently is considered a form of universal lipid signaling [41]. Generally, PA constitutes only a minor proportion of the cellular lipid pool, but in plants, in response to different stress factors, the PA level can increase significantly [42,43]. PA might act as a membrane-localized signal to recruit specific target proteins, including those involved in response to environmental stress. Upon binding, the PA–protein interaction changes protein leading, on one hand, to activation, but on the other hand, to the repression of the protein function [41]. Moreover, a greater loss of PC and increase in PA may be responsible for destabilizing the structure bilayer membrane, causing a greater propensity to membrane fusion and cell death [44].

The disruption of the membrane function, a reduction in its thickness and increased leakage have been attributed to structural changes related to modifications of membrane lipid composition and lipid peroxidation. Changes in cell membranes, especially an increase in the saturation of cell lipids, have been reported for plants treated with Al, Cd and Ni [45,46,47]. However, relatively little is known about Pb treatment’s implications for lipid metabolism in plant cells, and there are no data concerning the influence of exogenous melatonin on lipid composition of cell membranes.

The effects of melatonin pretreatment on cytotoxic properties of lead were determined on BY-2 suspension cells, which are an excellent model for examining plant physiology, biochemistry and molecular biology [48].

The objective of the present study was to examine if the pretreatment of suspension *Nicotiana tabacum* BY-2 cells with melatonin inhibits Pb-induced cell death, as well as determining whether exogenous melatonin may contribute to lipid profile modification and the limitation of the negative effects of this heavy metal on plant membrane composition. The obtained results could facilitate the understanding of lipid functions in plant cells exposed to Pb, as well as reveal if melatonin has a protective influence on biomembranes and acts as a biostimulating, pro-survival factor.

## 2. Results

### 2.1. BY-2 Growth and Mortality

The supplementation of BY-2 culture media with melatonin prior to lead exposure slightly improved cell proliferation and protected cells from death, especially in the *log* phase of growth.

**Figure 1:**
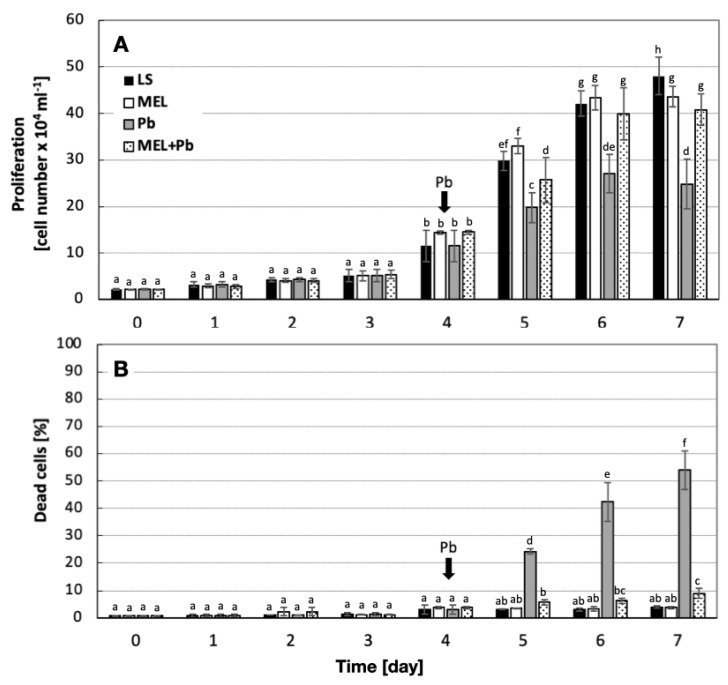
Kinetics of cell proliferation (**A**) and mortality (**B**) of BY-2 tobacco cells during in vitro culture (0–7 days). Cell variants: LS, MEL, Pb and MEL+Pb were prepared and treated according to the description included in Section 4. (**A**) Proliferation ANOVA results: variant (LS, MEL, Pb, MEL+Pb) F_(3;87)_ = 47.6 *p* < 0.000001; day of culture (0, 1, 2, 3, 4, 5, 6, 7) F_(7;87)_ = 765.9 *p* < 0.000001; interaction variant × day of culture F_(21;87)_ = 13.1 *p* < 0.000001. (**B**) Mortality ANOVA results: variant (C, MEL, Pb, MEL+Pb) F_(3;71)_ = 334.7 *p* < 0.00001; day of culture (0, 1, 2, 3, 4, 5, 6, 7) F_(7;71)_ = 165.6 *p* < 0.00001; interaction variant × day of culture F_(21;71)_ = 98.4 *p* < 0.00001. Statistically different values are shown by dissimilar letters.

The intensity of culture growth in LS and MEL variants was similar throughout the entire duration of the experiment. After adding lead on the 4th day to BY-2 suspension cells, significant inhibition of proliferation was observed (variant Pb). However, the proliferation of the MEL+Pb cells was about 40% higher in comparison to Pb samples. This effect remained until the end of the BY-2 cell culture (Figure 1A).

To check the influence of melatonin on cell mortality, parallel to cell proliferation, the level of cell viability was measured in all experimental samples. Methylene blue staining evidenced that the supplementation of culture medium with melatonin did not result in cell death acceleration. Moreover, the obtained results revealed a comparable level of cell viability between lead-untreated cells (LS and MEL). Surprisingly, lead-treated but primed with melatonin samples (MEL+Pb) had significantly higher levels of viability (Figure 1B). Received data showed that at the end of the *log* phase (the 7th day of culture; 72 h after lead administration), the viability of BY-2 cells in the MEL+Pb variant was about 85% higher in comparison to the PB variant (Figure 1B).

### 2.2. Red-Ox Status

**Figure 2:**
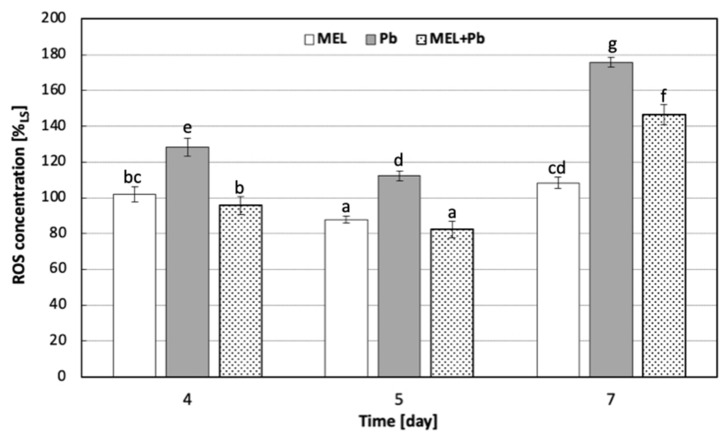
ROS concentration in all experimental variants of BY-2 tobacco cells exposed to lead. Cell variants: LS, MEL, Pb and MEL+Pb were prepared and treated according to the description included in Section 4. The intensities of DCF fluorescence levels were measured 4 h (4 d), 24 h (5 d) and 72 h (7 d) after lead treatment. The fluorescence of BY-2 control cells (LS) in the above-indicated moments of the experiment were assumed to be 100%. ROS accumulation [% of LS] ANOVA results: variant (MEL, Pb, MEL+Pb) F_(2;18)_ = 241.3 *p* < 0.000001; day of culture (4, 5, 7) F_(2;18)_ = 363.3 *p* < 0.000001; interaction variant × day of culture F_(4;18)_ = 38.8 *p* < 0.000001. Statistically different values are shown by dissimilar letters.

The effect of melatonin on ROS production in BY-2 suspension cells exposed to lead, expressed as DCF fluorescence intensity, is presented in Figure 2. Our results clearly demonstrate that lead generated ROS cumulation in BY-2 cells. Just 4 h after lead treatment, ROS fluorescence in the Pb variant was 30% higher than in the LS control samples. The highest ROS level was observed after 72 h of incubation of cells with heavy metal, and it was about 75% higher than in the control one. However, when cells exposed to lead were preincubated with melatonin, about 25%, 30% and 20% lower ROS production was detected at 4 h, 24 h and 72 h after lead administration, respectively, compared with the Pb variant. Thus, melatonin ameliorated lead’s pro-oxidative effect.

**Figure 3:**
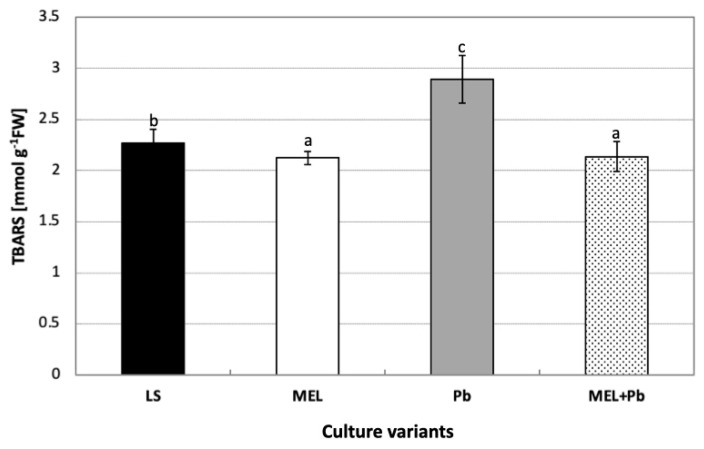
TBARS determination in BY-2 tobacco cells exposed to lead. Cell variants: LS, MEL, Pb and MEL+Pb were prepared and treated according to the description included in Section 4. Assays were performed 72 h (7 d) after lead treatment. TBARS ANOVA results: variant (LS, MEL, Pb, MEL+Pb) F_(3;25)_ = 15.5 *p* < 0.00001. Statistically different values are shown by dissimilar letters.

On the 7th day of the experiment, when the ROS level was the highest, the first effects of oxidative damage of plasma membranes were observed—Figure 3. The highest accumulation of TBARS was observed in the Pb variant, and it was about 23% and 13% higher for Pb vs. MEL+Pb and LS vs. MEL, respectively (Figure 3).

Thus, these observations demonstrated that melatonin preincubation protected tobacco suspension cells from lead-induced lipid peroxidation estimated via TBARS cumulation.

### 2.3. Characterization of Phospholipid Molecular Species

**Figure 4:**
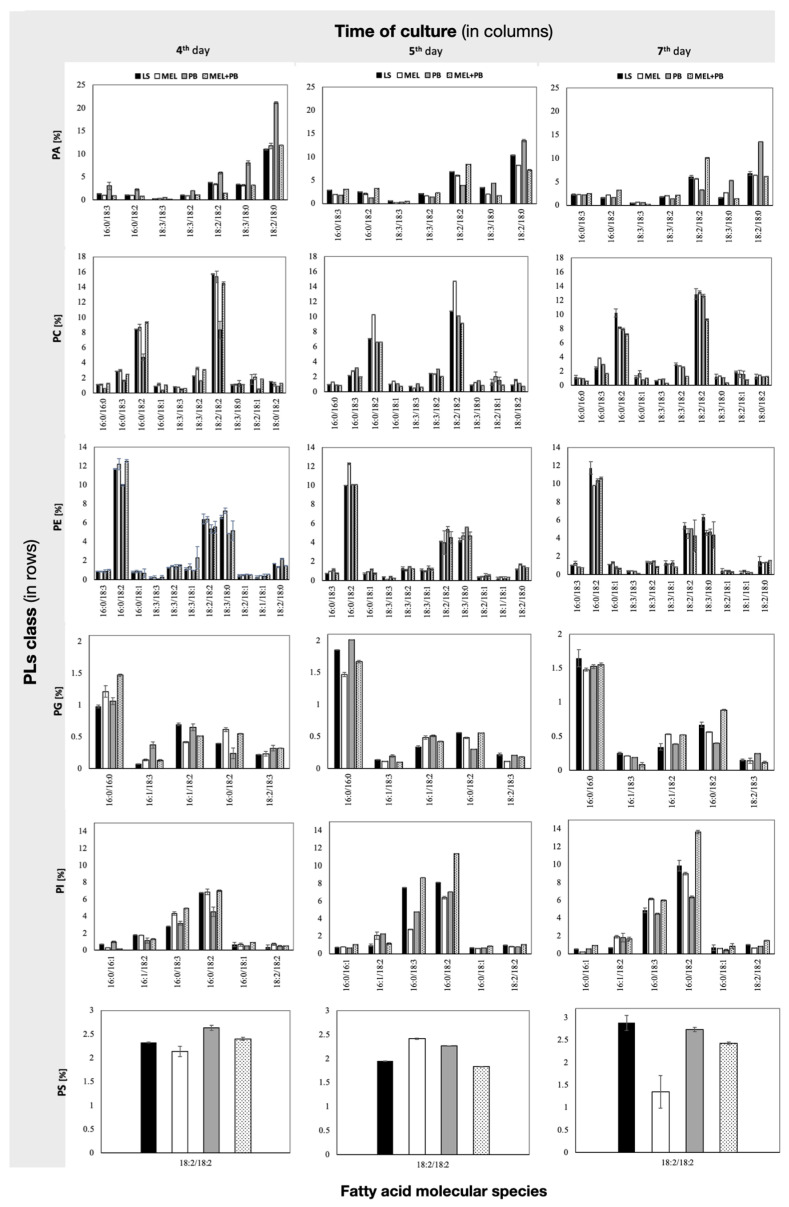
Panel presenting fatty acid composition in a particular class of phospholipids (PLs) in BY-2 tobacco cells exposed to lead. Cell variants: LS—BY-2 cells cultured on LS medium (the control variant); MEL—BY-2 cells cultured on LS medium with 200 nM of melatonin added from the beginning of the culture; PB—BY-2 cells cultured on LS medium with 15 µM Pb^2+^ added on the 4th day of the culture; MEL+PB—BY-2 cells cultured on LS medium with melatonin added from the start of the culture and with Pb^2+^ added on the 4th day of culture. PL classes in rows: PA—phosphatidic acid; PC—phosphatidylcholine; PE—phosphatidylethanolamine; PG—phosphatidylglycerol; PI—phosphatidylinositol; PS—phosphatidylserine. These classes were analyzed in cells 4 h (4th day column), 24 h (5th day column) and 72 h (7th day column) after lead treatment.

Lipidomic analysis of BY-2 cell extracts from all experimental variants revealed the presence of 40 PL types from six classes: PA, PC, PE, PG, PI and PS (Figure 4). The numbers, identified on the basis of fatty acid composition, of species in the above-mentioned classes were 7, 10, 11, 5, 6 and 1, respectively. In control and MEL cells, the main class of phospholipids, representing almost 40% of the total lipid content, was PC. PE and PA were the second and third classes in all analyzed time points.

**Figure 5:**
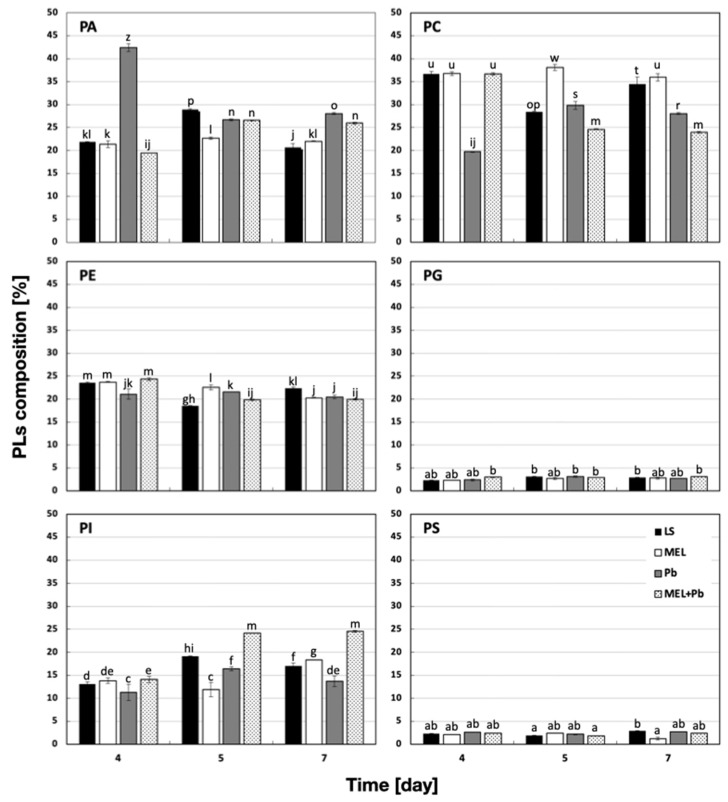
Comparison of phospholipid (PL) composition in BY-2 tobacco cells exposed to lead. Cell variants: LS—BY-2 cells cultured on LS medium (the control variant); MEL—BY-2 cells cultured on LS medium with 200 nM of melatonin added from the beginning of the culture; Pb—BY-2 cells cultured on LS medium with 15 µM Pb^2+^ added on the 4th day of the culture; MEL+Pb—BY-2 cells cultured on LS medium with melatonin added from the start of the culture and with Pb^2+^ added on the 4th day of culture. The panels present different classes of PLs: PA—phosphatidic acid; PC—phosphatidylcholine; PE—phosphatidylethanolamine; PG—phosphatidylglycerol; PI—phosphatidylinositol; PS—phosphatidylserine. They were estimated in cells 4 h (4 d), 24 h (5 d) and 72 h (7 d) after lead treatment. PLs composition ANOVA results: variant (LS, MEL, Pb, MEL+Pb) F_(3;144)_ = 0.2 *p* = 0.90; PLs (PA, PC, PE, PG, PI, PS) F_(5;144)_ = 20168 *p* < 0.000001; day of culture (4, 5, 7) F_(2;144)_ = 0.3 *p* = 0.76; and interactions variant × PLs F_(15;144)_ = 359.6 *p* < 0.000001; variant × day of culture F_(6;144)_ = 0.043 *p* = 0.99; PLs × day of culture F_(10;144)_ = 126.2 *p* < 0.000001; variant × PLs × day of culture F_(30;144)_ = 198.3 *p* < 0.000001. Statistically different values are shown by dissimilar letters.

Our results showed a drastic reduction in the PC level just 4 h after lead treatment, whereas twice as much PA was detected in comparison to the control or melatonin-supplemented samples (LS, MEL, MEL+Pb) (Figure 5).

Although during the culture period, the cells adjusted to the stress and reduced the level of PA, on the last day, it was still 10% higher than in cells protected with melatonin. At the end of culture, the level of PA in Pb samples was the highest among all experimental variants, but priming with melatonin decreased PA by about 10% (see MEL+Pb vs. Pb).

**Figure 6:**
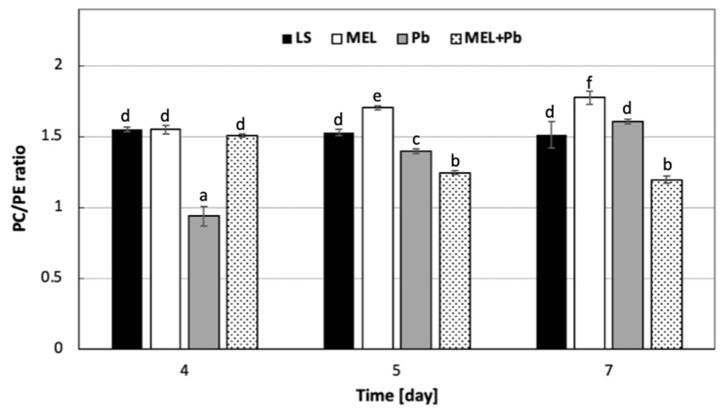
The changes in the PC/PE ratio in BY-2 tobacco cells exposed to lead. Cell variants: LS—BY-2 cells cultured on LS medium (the control variant); MEL—BY-2 cells cultured on LS medium with 200 nM of melatonin added from the beginning of the culture; Pb—BY-2 cells cultured on LS medium with 15 µM Pb^2+^ added on the 4th day of the culture; MEL+Pb—BY-2 cells cultured on LS medium with melatonin added from the start of the culture and with Pb^2+^ added on the 4th day of culture. Phospholipids: PC—phosphatidylcholine; PE—phosphatidylethanolamine. They were estimated in cells 4 h (4 d), 24 h (5 d) and 72 h (7 d) after lead treatment. PC/PE ratio ANOVA results: variant (LS, MEL, Pb, MEL+Pb) F_(3;24)_ = 247.6 *p* < 0.000001; day of culture (4, 5, 7) F_(2;24)_ = 49.3 *p* < 0.000001; interaction variant × day of culture F_(6;18)_ = 113.8 *p* < 0.000001. Statistically different values are shown by dissimilar letters.

It should be pointed out that on membrane function, the modification of the PC/PE ratio also has influence. Our data showed that the PC/PE ratio significantly dropped after lead administration. During 4 h of cell culture with lead, the PC/PE ratio was about 60% lower in comparison to the lead-untreated samples LS and MEL. Interestingly, in MEL+Pb probes, at the time of culture, the PC/PE ratio was similar to those obtained for unstressed cells (LS and MEL) (Figure 6).

On the contrary, the prolongation of culture to the stationary phase of growth resulted in the increase in PA in Pb samples without changes in PC and PE levels and, consequently, kept the PC/PE ratio similar to LS. Surprisingly, in the MEL+Pb variant, the PC/PE ratio was about 25% lower in comparison to melatonin-untreated cells (Pb) (Figure 5 and Figure 6). In the MEL variant, a significant increase in the PC/PE ratio was observed. The cells tried to rebuild the lowering of the PC/PE ratio on the 5th and 7th days to the control level, while the surprising decrease in the ratio in the MEL+Pb variant can be seen in the fact that MEL activated a completely different membrane defense strategy, i.e., it caused the greatest increase in the PI content in the PLS pool. It is known that PI is important for growth and metabolism; therefore, important results were obtained in MEL+Pb variants (Figure 5 and Figure 6). During all culture periods in MEL+Pb probes, the highest PI content was observed. Comparing the PI proportion between Pb and MEL+Pb, we observed about 20%, 30% and 50% lower PI levels at 4 h, 24 h and 72 h after lead exposition, respectively. In Figure 5, when comparing PI and PS levels in the samples, we can observe that higher levels of PI were negatively correlated with slightly lower PS levels.

The metabolomic analysis of BY-2 extracts identified saturated, monounsaturated and polyunsaturated fatty acid. The distribution of the hydrocarbon chains showed that palmitic (C16:0), palmitoleic (C16:1) and stearic (C18:0) were the most predominant saturated fatty acids, whereas oleic (C18:1), linoleic (C18:2) and γ-linolenic (C18:3) were the major unsaturated acid species (Figure 4). The incubation of BY-2 cells for 4h with lead (Pb) increased the contents of most phospholipid species containing C 18:3: and C 18:2: PA 16:0/18:3, PA 16:0/18:2, PA 18:3/18:3, PA 18:3/18:2, PA 18:2/18:2, PA 18:3/18:0, PA 18:2/18:0, PE 18:2/18:0, PG 16:1/18:3, PG 18:2/18:3 and PS 18:2/18:2. These results were accompanied by declines in almost all of them when BY-2 suspension cells were preincubated with melatonin prior to lead. On the other hand, in Pb samples, we observed drastic declines in the amounts of PC 16:0/16:0, PC 16:0/18:3, PC 16:0/18:2, PC 18:3/18:2, PC 18:2/18:2, PC 18:2/18:1, PE 16:0/18:2, PE 18:3/18:3, PE 18:3/18:0, PG 16:0/18:2 and PI 16:0/18:2 but reverse effects in MEL+Pb probes (Figure 4). On the 5th day, elevated levels of phospholipid species were observed mainly for LS and MEL samples, but the incubation of BY-2 cells with lead only slightly changed the percentage compositions of phospholipid species. The prolongation of BY-2 culture to the stationary phase of growth (day 7th, 72 h after lead treatment) resulted in increasing three times and twice the levels of PA 18:3/18:0 and PA 18:2/18:0 in comparison to the melatonin-supplemented variant (MEL+Pb), respectively (Figure 4).

**Figure 7:**
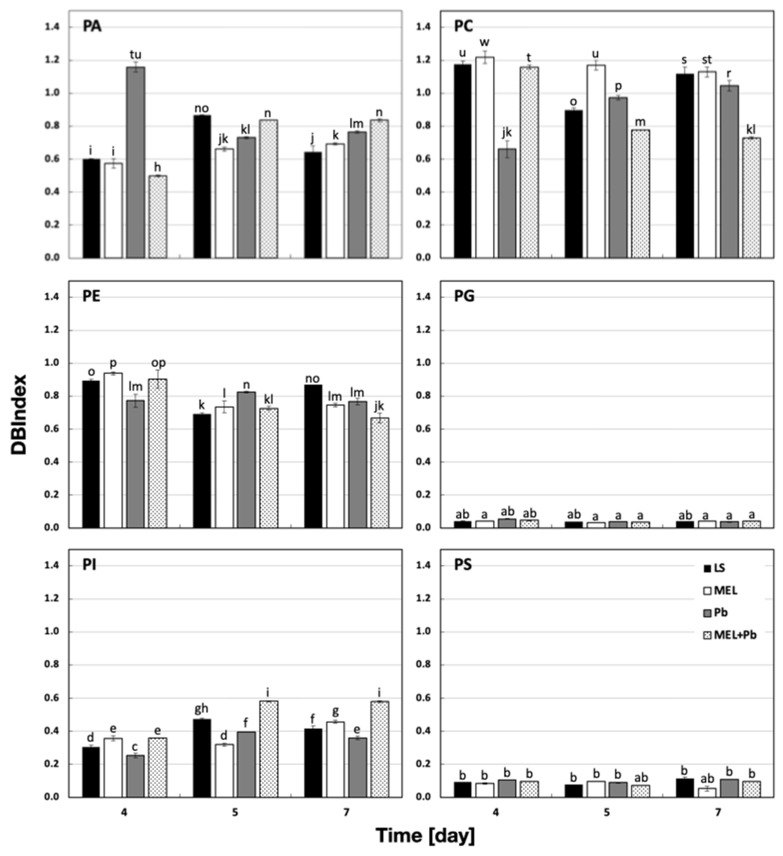
The changes in double-bond indexes (DBIndexs) of phospholipids (PLs) isolated from BY-2 tobacco cells exposed to lead. Cell variants: LS—BY-2 cells cultured on LS medium (the control variant); MEL—BY-2 cells cultured on LS medium with 200 nM of melatonin added from the beginning of the culture; Pb—BY-2 cells cultured on LS medium with 15 µM Pb^2+^ added on the 4th day of the culture; MEL+Pb—BY-2 cells cultured on LS medium with melatonin added from the start of the culture and with Pb^2+^ added on the 4th day of culture. Panels present PLs: PA—phosphatidic acid; PC—phosphatidylcholine; PE—phosphatidylethanolamine; PG—phosphatidylglycerol; PI—phosphatidylinositol; PS—phosphatidylserine. They were estimated in cells 4 h (4 d), 24 h (5 d) and 72 h (7 d) after lead treatment. DBIndex ANOVA results: variant (LS, MEL, Pb, MEL+Pb) F_(3;72)_ = 8.7 *p* < 0.0001; PLs (PA, PC, PE, PG, PI, PS) F_(5;72)_ = 12203 *p* < 0.000001; day of culture (4, 5, 7) F_(2;72)_ = 4.7 *p* < 0.05; and interactions variant × PLs F_(15;72)_ = 137.4 *p* < 0.000001; variant × day of culture F_(6;72)_ = 6 *p* < 0.00005; PLs × day of culture F_(10;72)_ = 73.6 *p* < 0.000001; variant × PLs × day of culture F_(30;72)_ = 110.9 *p* < 0.000001. Statistically different values are shown by dissimilar letters.

To identify the possible role of phospholipid fatty acids in lead tolerance after melatonin supplementation, a double-bond index (DBIndex), which indicates the level of lipid unsaturation, was used. A higher DBIndex points to lower saturation of fatty acids. The most evident changes were revealed at the beginning of the logarithmic phase of growth. In Pb samples, 4 h after lead administration, the DBIndex for PA was 90% higher in comparison to the control. The amounts of more unsaturated fatty acids significantly decreased when cells were primed with melatonin (MEL+Pb). The obtained results indicated a decrease in the DBIndex by about 60% for PA. On the other hand, when cells were incubated with lead and not primed with indoleamine, a drastic drop in the DBIndex value was observed for PC. Lead induced an about 2-fold decrease in the DBIndex for PC; however, melatonin supplementation (MEL+Pb) elevated the DBIndex values to match those detected in control cells (Figure 7).

The prolongation of BY-2 cell treatment with lead to the stationary phase of growth caused increasing DBIndex values for PA, which was in line with higher amounts of less saturated fatty acids, especially C 18:2 (Figure 4 and Figure 7).

### 2.4. Phospholipase D Activity

**Figure 8:**
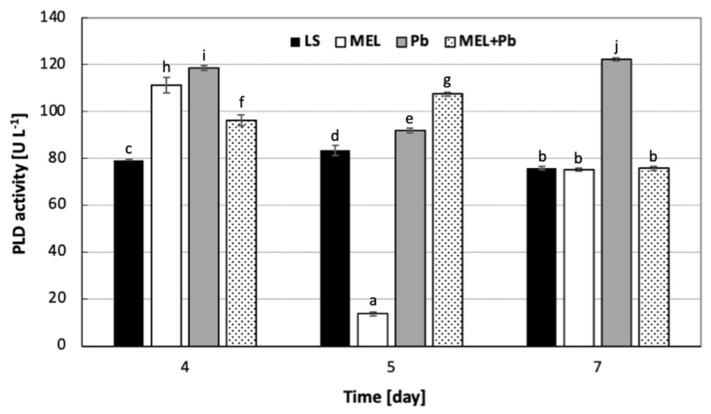
The changes in phospholipase D activity in BY-2 tobacco cells exposed to lead. Cell variants: LS—BY-2 cells cultured on LS medium (the control variant); MEL—BY-2 cells cultured on LS medium with 200 nM of melatonin added from the beginning of the culture; Pb—BY-2 cells cultured on LS medium with 15 µM Pb^2+^ added on the 4th day of the culture and MEL+Pb—BY-2 cells cultured on LS medium with melatonin added from the start of the culture and with Pb^2+^ added on the 4th day of culture. Enzyme activities were measured with Phospholipase D Assay Kit (Sigma-Aldrich; Saint Louis; USA) 4 h (4 d), 24 h (5 d) and 72 h (7 d) after lead treatment. PLD activity ANOVA results: Variant (LS, MEL, Pb, MEL+Pb) F_(3;24)_ = 1475 *p* < 0.000001; Day of culture (4, 5, 7) F_(2;24)_ = 1015 *p* < 0.000001; and interaction Variant × Day of culture F_(6;18)_ = 1024 *p* < 0.000001. Statistically different values are shown by dissimilar letters.

To complete research on the changes in PLs’ compositions, we also checked the activity of PLD, which hydrolyzes PC or PE and contributes mostly to the formation of PA. Figure 8 illustrates the effects of melatonin and Pb^2+^ treatments on PLD activity in BY-2 cells in all experimental variants.

In the logarithmic phase of growth, 4 h after lead addition (Pb), cells demonstrated about 50% higher PLD activity than the control once. Surprisingly, 24 h after lead exposition, a drastic decrease in PLD was observed in the MEL variant. At this experimental time-point, the highest activity of PLD was noticed for the MEL+Pb variant, and it was nearly 8-fold higher than in cells primed with melatonin and not exposed to lead. At the stationary phase of growth (7 d), we found a 60% increase in PLD activity for the Pb variant, whereas in the LS, MEL and even MEL+Pb samples, enzyme action was at a similar level.

## 3. Discussion

Efficient metabolism depends on the proper functioning of biological membranes. At the cytological level, they are a sensitive element determining the plant’s resistance to abiotic stresses. Therefore, researchers’ attention is directed towards explaining the mechanisms that stabilize their structure and functioning, especially in unfavorable environmental conditions. Membrane lipids are of particular interest since variations in their content are known to determine the maintenance of membrane integrity [49]. Moreover, they provide the successful execution of protective, structural, transport and signaling functions by the membranes, as well as participate in the regulation of membrane-bound protein actions [44]. Our previous studies revealed that melatonin is an excellent biostimulant that fortifies cells against potentially harmful conditions and limits their mortality [17,18]. This highly amphiphilic indoleamine readily crosses cellular membranes and exhibits high antioxidant effectiveness and free radical scavenging ability [20]. Moreover, our results indicated that BY-2 cells were able to synthesize this molecule depending on the growth phase, as well as uptake it actively from the medium to prevent ROS accumulation after lead treatment [18].

Lead is a non-essential chemical element but easily absorbed by the plant. Its toxicity negatively effects water and hormonal status and alters carbohydrate metabolism and enzyme activity, as well as the uptake and distribution of minerals [50]. Heavy metal toxicity to living cells can be also manifested by an increase in membrane permeability, leading to disruption in their function. However, the mechanism involved in heavy metal-induced dysfunctions is not fully understood. Hasanuzzaman et al. [51] reported the disruption of antioxidant defense and redox balance after Ni in *Oryza sativa* seedlings. Cd, Cu and Hg promoted enhanced membrane permeability and generated ROS in mitochondria [52]. Nahar et al. [53] documented that Al induced oxidative damage, overproducing ROS and increasing lipoxygenase activity. Additionally, Zn can interrupt the synthesis of lipids and induced rise in fatty acid unsaturation [54]. Kawai-Yamada et al. [55] reported that oxidative metabolism leading to the generation of ROS is one of the earliest events in programmed cell death (PCD) induced by biotic or abiotic stress in tobacco plants. On the other hand, it is known that common hallmarks of animal and plant PCD are changes in plasma membrane, including a decrease in membrane potential and its disintegration [56].

Thus, in the present study, the effects of melatonin on ROS production, lipid profile modification and the limitation of the negative effects of lead on membrane composition in BY-2 suspension cells were analyzed. Our results clearly demonstrate that lead generated ROS, increasing in BY-2 cells. Just 4 h after lead treatment, ROS fluorescence in the Pb variant was 30% higher than in the control samples. The highest ROS level was observed after 72 h of incubation of cells with heavy metal, and it was about 75% higher than in the LS variant. Interestingly, when BY-2 cells were exposed to lead but preincubated with melatonin, about 20–30% lower ROS production was detected in comparison to Pb samples. What was important was that incubation cells with melatonin alone did not elevate ROS levels. Reduced ROS levels in MEL+Pb cells were accompanied by 40% higher cell proliferation and over 80% higher viability.

Due to the fact that the highest level of ROS was observed in samples 72 h after lead administration, the index of lipid peroxidation, widely used as an indicator of ROS mediated damage to cell membranes under stressful conditions, was checked [57]. To investigate the level of lipid peroxidation, TBARS content was measured 72 h after lead exposition in all experimental variants. It is well known that an imbalance between the generation of ROS and their removal via an antioxidant system implies the induction of oxidative stress, leading to lipid peroxidation and even cell death. However, there are some papers indicating no evidence of lipid peroxidation after Cd or Ni treatment, although the accumulation of ROS was observed [47,58,59]. Labudda et al. [60] even showed nearly two-fold lower TBARS content after Cd exposure. Taking these data into account, it seems that apart from lipid peroxidation, changes in lipid composition might also contribute to membrane damage. Considerable enhancement of lipid peroxidation was found only during longer exposure to heavy metals or their presence in unusually high concentrations [61].

The prolongation of BY-2 cell culture to the stationary phase of growth (day 7th—72 h after Pb-treatment) revealed that TBARS level was about 23% and 13% higher for Pb vs. MEL+Pb and Pb vs. LS, respectively. Thus, these observations demonstrated that the preincubation of tobacco suspension cells with melatonin protected them from lead-induced lipid peroxidation and TBARS accumulation, especially during prolonged culture.

There is a limited number of studies in the published literature related to the interaction of melatonin in cell membranes at the molecular level. The available mechanistic information comes mainly from studies of lipid vesicles (micelles) containing one or several phospholipids. Data revealed by Ceraulo et al. [62] showed that melatonin is deposited preferentially in an external position in the lipid layers of the cell membrane near the polar heads of these molecules. Its juxtaposition to the lipid molecules allows this indoleamine to defend them from the attacks of free radicals. The lipid-protective actions of melatonin have been proven in numerous human diseases, and they are probably connected to the fact that among subcellular organelles, membranes have high intrinsic levels of melatonin [63,64]. Moreover, it is known that both endogenously generated and exogenously administered melatonin have important roles in membrane component remodeling, consequently reducing membrane rigidity and preserving optimal membrane fluidity in mammals [64,65]. In the plant kingdom, the differences in membrane composition especially in membrane phospholipids were detected in the responses of cold [44]; Ni, Cd and Cu [47,54]; and salinity stress [66].

Lipidomic analysis of BY-2 cells in optimal (LS), melatonin-supplemented (MEL) and lead treatment conditions with and without melatonin (MEL+Pb and Pb, respectively) revealed the presence of 40 types of PL from six classes: PA, PC, PE, PG, PI and PS, and the number of identified species were, respectively, 7, 10, 11, 5, 6 and 1. In the control and MEL variants, the main classes of phospholipids were PC and PE. Because PC and PE play a crucial role in the maintenance of proper fluidity and permeability of the membrane, their variability seems to be particularly important [54,67]. Our results showed drastic a reduction in the PC level just 4 h after lead treatment, whereas twice as much PA was detected in comparison to the control or melatonin-supplemented samples (LS, MEL, MEL+Pb). At the end of culture, the level of PA in Pb samples was the highest among all the experimental variants. Usually, PA constitutes only a small part of the cellular lipid pool, but in plants, in response to different stress conditions, such as pathogens, ROS, chilling, salts, injuries and heavy metal exposure, PA levels may increase significantly [4,37,41,68]. PA might act as a membrane-localized signal to recruit specific target proteins, including those involved in the response to environmental stress. Upon binding, the PA–protein interaction changes protein leading, on one hand, to the activation but, on the other hand, to the repression of the protein function [41].

The excess formation of PA mostly contributes to the hydrolysis of structural membrane phospholipids, such as PC and PE, by PLD. Because unsaturated PEs have a strong propensity to form hexagonal phases and PA has a tendency to form a hexagonal II phase in the presence of Ca^2+^, these compositional alterations are likely to lead to the formation of a non-bilayer lipid phase. The greater loss of PC and increase in PA may be responsible for destabilizing the bilayer membrane structure, resulting in a greater tendency for membrane fusion and cell death [44].

The exposure of BY-2 cell culture for 4 h to lead reduced the PC/PE ratio by about 60% in comparison to the lead-untreated samples LS and MEL. Moreover, we observed that at the time of culture, the PC/PE ratio in MEL+Pb probes was similar to those obtained for unstressed cells (LS and MEL). In contrast, the prolongation of culture to the stationary phase of growth resulted in keeping the PC/PE ratio at a similar or lower level in comparison to LS, respectively, for Pb and MEL+Pb. In the MEL+Pb variant, the PC/PE ratio was about 25% lower in comparison to melatonin-untreated cells (Pb), which was probably connected to the decline in the PC and PE contents. However, in both these cases (Pb and MEL+Pb), the accumulation of PA was noted. Thus, PLD activity in all experimental variants was checked. Cells 4 h after lead addition (Pb) demonstrated about 50% higher PLD activity than the control once. These effects were accompanied with findings that the higher enzyme activity resulted in PA accumulation, as well as a reduction in PC. On the contrary, at the stationary phase of growth, the accumulation of PA in Pb, as well as in MEL+Pb variants, was observed, although we found a 60% increase in PLD activity only in Pb probes. In MEL+Pb samples, the enzyme action was similar to those detected for LS and MEL. The literature data indicate that PA can also be generated by the sequential action of phospholipase C (PLC) and diacylglycerol kinase (DGK) [69,70]. Nevertheless, PLC/DGK pathway-mediated PA formation decreases upon the disruption of phosphatidylinositol 4-phosphate (PtdIns(4)P) and phosphatidylinositol 4,5-bisphosphate (PtdIns(4,5)P2) biosynthesis, which show that the activity of phosphoinositide-specific phospholipase C (PI-PLC) is dependent on its substrates PtdIns4P and PtdIns(4,5)P2 [71]. These results are in line with our observations indicating that the accumulation of PA in parallel with a decreasing PLD level result in elevation of about 50% of PI—a key molecule required to obtain the derivatives PtdIns(4)P and PtdIns(4,5)P2.

Changes in cell membranes, especially increases in the saturation of cell lipids, have been reported for plants treated with Al, Cd and Ni [45,46,47]. However, relatively little is known about Pb^2+^ treatment’s implication for lipid metabolism in plant cells, and there is no data concerning the influence of the exogenous melatonin on the lipid compositions of cell membranes.

Analysis of the distribution of the hydrocarbon chains showed that palmitic (C16:0), palmitoleic (C16:1) and stearic (C18:0) were the most predominant saturated fatty acids, whereas oleic (C18:1), linoleic (C18:2) and γ-linolenic (C18:3) were the major unsaturated acid species. The incubation of BY-2 cells for 4 h with lead increased the contents of most phospholipid species containing C 18:3: and C 18:2: PA 16:0/18:3, PA 16:0/18:2, PA 18:3/18:3, PA 18:3/18:2, PA 18:2/18:2, PA 18:3/18:0, PA 18:2/18:0, PG 16:1/18:3, PG 18:2/18:3 and PS 18:2/18:2. These results were accompanied by declines in almost all of them when BY-2 suspension cells were preincubated with melatonin. On the other hand, in PB samples, we observed drastic declines in PC 16:0/18:3, PC 16:0/18:2, PC 18:3/18:2, PC 18:2/18:2, PC 18:2/18:1, PE 18:3/18:3, PG 16:0/18:2 and PI 16:0/18:2. Priming BY-2 cells with melatonin prior to lead administration elevated the abundance of these species to the control level. Opposite changes in phospholipid molecular species were observed by Bernat et al. [37] after Ni treatment of wheat seedlings; among the analyzed phospholipids, PA 16:0/18:3, PA 16:0/18:2 and PA 18:3/18:3 significantly decreased, whereas only PA 18:2/18:2 rose.

Unsaturated fatty acids play important roles in the structure and functioning of cell membranes. Indeed, the degree of saturation impacts not only the physicochemical characteristics of the fatty acids, such as the melting point or the viscosity, but also the molecular shape of the lipid to which it is attached. This, in turn, influences the membrane lipid behavior and the bilayer packing [72]. In addition, the unsaturated level of fatty acids in plants may vary, at least in a certain range, in response to different environmental situations; therefore, a DBIndex, which indicates the level of lipid unsaturation, was checked. Higher DBIndex points to the lower saturation of fatty acids. The most evident changes were revealed in the logarithmic phase of growth, and the prolongation of lead treatment showed no spectacular results. The DBIndex for the Pb variant compared to the control was 90% and 40% higher in PA and PG, respectively. On the other hand, a drastic decline in the DBIndex value was observed for PC. Changes in the composition of fatty acids, resulting in an increase in saturation, have been reported for plants subjected to cold, draught, salinity and heavy metals [73]. According to Chaffai et al. [45], a more saturated fatty acid composition may result in increased membrane stiffness and, consequently, in the restriction of the entry of toxic metal ions to plant cells, which can be an adaptation mechanism during heavy metal stresses. Thus, a decrease in polyunsaturated fatty acid content may protect membranes against severe damage and maintain proper permeability. However, Leekumjorm et al. [74] proved that the presence of unsaturated fatty acids was necessary to maintain appropriate membrane fluidity and the preservation of the uniform level of its hydration, which ensured the stabilization of membranes. Therefore, a decrease in the unsaturation level may destabilize membranes and affect their integrity. In our work, an increased DBIndex after lead treatment was mainly caused by the elevation of PA containing C18:3 and C18:2. These observations are in accordance with those reported by Słaba et al. [39] that the toxic action of lead was connected to an enhanced propidium iodide influx, indicating a strong membrane permeabilization. However, priming BY-2 cells with melatonin (MEL+Pb) resulted in changing the DBIndex to a level similar to those of lead-untreated samples. These effects suggest that the supplementation of culture media with melatonin diminishes the membrane perturbation. Nevertheless, the decreased unsaturation appears to be important because polyunsaturated acids are particularly sensitive to attack by ROS and peroxidation. This process can lead to chain breakage and, thereby, increases in membrane fluidity and permeability.

Considering the received data, we can clearly conclude that lead exposition changes phospholipids’ profiles in tobacco BY-2 cell culture, resulting in membrane remodeling and disruption, but exogenous melatonin modifies obtained outlines to match those without heavy metal stress. However, it should be remembered that our experiments were conducted in an in vitro cell culture of the model tobacco species BY-2. Such conditions allow us to relatively quickly metabolomically select a new field of melatonin actions in the context of counteracting Pb stress and further investigate molecular aspects of this phenomenon, e.g., signal transduction conducted due to these positive effects. However, similar studies on whole plants in vivo should not be omitted in the future, because only these kind of experiments can be conducted to assess MEL’s practical use in horticulture or agriculture.

## 4. Materials and Methods

### 4.1. Plant Material

In experiments, the sterile suspension of the in vitro cell culture of *Nicotiana tabacum*, L. cv Bright Yellow 2 (BY 2) was used. Tobacco BY 2 cells were cultivated on Linsmaier and Skoog basal medium (LS) [75] in Nagata modification [76].

The pH of the medium was adjusted to 5.3.

### 4.2. Experimental Treatments

BY-2 cells at the stationary growth phase of growth (day 7th) were transferred into the fresh LS medium in the following variants:

(i) LS (optimal culture conditions—control);
(ii) MEL (LS medium with 200 nM of melatonin added from the beginning of culture);
(iii) Pb (LS medium with Pb^2+^ added on the 4th day of culture)
(iv) MEL+Pb (LS medium with melatonin added from the beginning of culture and stressed with Pb^2+^ added on the 4th day of culture).

The lead concentration used in our experiments was chosen after the measurement of LC_50_ on the 7th day of BY-2 cell culture. The optimal dose of melatonin was previously chosen experimentally. To choose an efficient MEL concentration, we took under consideration two aspects: (1) MEL should not affect cell proliferation; (2) it should reverse Pb toxicity. MEL in concentrations lower than 200 nM did not reversed Pb toxicity significantly. MEL in concentrations higher than 200 nM mitigated BY-2 cell proliferation. We also observed the effects of MEL supplementation from the start of culture and from the logarhitmic phase of growth (to select best timing). MEL added from the start of culture (first day) acted more effectively and fortified cells for heavy metal stress. Thus, both Pb and MEL concentrations were chosen experimentally according to preliminary tests.

### 4.3. Determination of Cell Growth and Viability

The cell number was determined under a light microscope after selective staining with methylene blue, with the use of a Fuchs–Rosenthal haemocytometer. Cells’ proliferation, as well as their viability, were analyzed every experimental day. The morphology of cells was examined using an Olympus CX-31 light microscope equipped with the MicroScan v.15. digital system of image analysis.

### 4.4. Measurements of Reactive Oxygen Species Generation

The generation of ROS was measured via DCFH_2_-DA assay [77,78]. Tobacco By-2 cells were seeded on 96-well black plates in amounts of 2 mg cells/well. The fluorescence of DCF was measured at 530 nm after excitation at 485 nm (DCFH_2_-DA, after deacetylation to DCFH_2_, is oxidized intracellularly to its fluorescent derivative, DCF). Assays were performed in modified Hank’s buffered salt solution (HBSS). The ROS content was measured on the 4th, 5th and 7th days of the experiment—4, 24 and 72 h after lead administration, respectively.

### 4.5. TBARS Measurement

The degree of lipid peroxidation was measured in categories of thiobarbituric acid-reacting substance (TBARS) contents according to the previously described protocol [17,79]. The TBARS content was calculated according to malondialdehyde (MDA) extinction coefficient of 155 mM^−1^ cm^−1^ and expressed as µmol MDA per g fresh weight (FW). The TBARS content was measured 72 h after lead administration (the 7th day of experiment).

### 4.6. Lipid Extraction Protocol

BY-2 cells were centrifugated at 12,000× *g* for 5 min. Next, 100 mg fresh weight was homogenized in a homogenizer (MP Biomedicals) with 1 mL of the homogeneous mixture containing methanol (2:1, *v*/*v*) and 0.003% BHT. After centrifugation at 4730× *g* for 5 min, the homogenate was vortexed with 0.2 mL of water and centrifuged again at 4730× *g* for 5 min. After transfer and evaporation of the organic phase, the residue was then redissolved in 1 mL of methanol/chloroform mixture (4:1, *v*/*v*) and stored at −20 °C for further analysis.

### 4.7. Phospholipid HPLC–MS Separation and Characterization of Its Molecular Species

Polar lipids were measured using an Agilent 1200 HPLC system (Santa Clara, CA, USA) and a 4500 Q-TRAP mass spectrometer (Sciex, Framingham, MA, USA) with an ESI source. For the reversed-phase chromatographic analysis, 10 μL of the lipid extract was injected onto a Kinetex C18 column (50 mm × 2.1 mm, particle size: 5 μm; Phenomenex, Torrance, CA, USA) with a flow rate of 500 μL min^−1^. The mobile phases water (A) and methanol (B) consisted of 5 mM ammonium formate. The solvent gradient was initiated at 70% B, increased to 95% B over 1.25 min and maintained at 95% B for 6 min before returning to the initial solvent composition over 3 min. The column temperature was maintained at 40 °C. The following mass spectrometer settings were applied: a spray voltage of −4.500 V, curtain gas 25, nebulizer gas 50, turbo gas 60 and an ion source temperature of 600 °C. The data analysis was performed with the Analyst™ v1.6.2 software (Sciex, Framingham, MA, USA). The phospholipids were determined qualitatively according to the methods described earlier [43]. Then, using a phospholipid standard for each PL class, phosphatidic acid (PA 12:0/12:0), phosphatidylcholine (PC 14:0/14:0), phosphatidylethanolamine (PE 14:0/14:0), phosphatidylglycerol (PG 14:0/14:0), lysophosphatidylcholine (LPC 16:0) and phosphatidylinositol (PI 16:0/16:0), a quantitate method was prepared. Analyses were performed on the 4th, 5th and 7th days of experiment—4, 24 and 72 h after lead administration, respectively.

The double-bond index (DBI), which indicates the unsaturation level of lipids, was calculated by the equation DBI = [sum of (N ×% lipid molecular species)]/100, where N is the number of double bonds in each lipid molecular species and % refers to the percentage of a complex lipid class [80].

### 4.8. Phospholipase D Activity Assay

Enzyme activity was measured using the Phospholipase D Activity Assay Kit (Sigma). In the used assay, phospholipase D (PLD) hydrolyzed PC to choline, which was determined using choline oxidase, resulting in a colorimetric (570 nm) product proportional to the PLD activity in the sample. Unit definition: one unit of PLD catalyzes the formation of 1 mM of choline per minute under the assay conditions (pH 7.4). Detailed analysis was carried out according to the manufacturer’s instructions. Extractions for analyzing PLD activity were performed by tobacco BY-2 cell (100 mg) homogenization in Cellytic buffer (Sigma) and then centrifugation at 4500× *g* for 5 min. The obtained supernatants were immediately tested. The calibration curve was prepared according to the manufacturer’s information for colorimetric detection. The PLD activity was measured on the 4th, 5th and 7th days of the experiment—4, 24 and 72 h after lead administration, respectively.

### 4.9. Statistical Analysis

All experiments were performed In at least physiological triplicate, with three test repetitions of each in the case of biochemical assays. The results are presented as their mean values (n = 9), and the error bars in the figures represent the standard deviation (±SD). Appropriate multivariate analysis of variance (ANOVA) and then post hoc Duncan multiple range tests were carried out to find the significant differences at *p* < 0.05. Statistically significant differences were marked on graphs with different small letters. The analysis was performed using the software STATISTICA ver. 13.0 (StatSoft).

## 5. Conclusions

Our results indicate that the pretreatment of BY-2 with exogenous melatonin protected tobacco cells against membrane dysfunction caused by oxidative stress (lipid oxidation), but also findings at the molecular level suggest a possible role for this indoleamine in protecting the lipid composition of the cell membrane, thus limiting lead-induced risk of cell death. The presented research indicates a new mechanism of the defense strategy of plant cells generated by melatonin. We would like our metabolomics studies of the model species to be an impact for similar *in vivo* studies on cultivated plants.

## Data Availability

Data is contained within the article.
